# Clinicopathologic characteristics and predictors of treatment uptake among patients with cervical cancer managed at Jimma University Medical Center, Southwest Ethiopia

**DOI:** 10.1186/s12885-026-15987-3

**Published:** 2026-04-14

**Authors:** Demisew Amenu Sori, Rahma Ali, Nega Jibat, Wondimagegn Adissu, Gerezgiher Buruh, Haftamu Hailekiros, Endashaw Nadew, Ashenafi Alemu, Alemseged Abdissa, Gelila K. Goba, Suzanne M. Garland, Nigisti Mulholland, Kim Mulholland, Eva J. Kantelhardt

**Affiliations:** 1 Department of Gynecology and Obstetrics, Faculty of Medical Science, Jimma University, Jimma, Ethiopia; 2 Institute of Medical Epidemiology, Biometry and Informatics, Global Health Working Group, Martin-Luther-University Halle- Wittenberg, Halle, Germany; 3 Department of Population and Family Health, Faculty of Public Health, Jimma University, Jimma, Ethiopia; 4 Department of Sociology, College of Social Science and Humanities, Jimma University, Jimma, Ethiopia; 5 School of Medical Laboratory, Faculty of Health Science, Jimma University, Jimma, Ethiopia; 6 Nursing Department, Mekelle University, Mekelle, Ethiopia; 7 Department of Medical Microbiology and Immunology, Mekelle University, Mekelle, Ethiopia; 8 Department of Communicable and Non-communicable Diseases, HIV and other Viral diseases research division, Armauer Hansen Research Institute, Addis Ababa, Ethiopia; 9 Department of Bacterial Viral Disease Research, Armauer Hansen Research Institute, Addis Ababa, Ethiopia; 10 Microbiologist, Armauer Hansen Research Institute, Addis Ababa, Ethiopia; 11 Department of Obstetrics and Gynecology, University of Miami, Coral Gables, Miami, USA; 12 Department of Obstetrics and Gynecology, University of Melbourne, Parkville, Melbourne, Australia; 13 Family and Reproductive Rights Education Program, Medical Sociologist, Melbourne, Australia; 14 Department of Pediatrics, University of Melbourne, Murdoch Children’s Research Institute, Parkville, Melbourne, Australia; 15 London School of Tropical Medicine, London , UK; 16 Global and Planetary Health Working Group, Department of Gynaecology, Medical Faculty of Martin-Luther-University Halle-Wittenberg, Halle, Germany

**Keywords:** cervical cancer, clinicopathology, outcomes, cervical cancer treatment, cervical cancer screening

## Abstract

**Background:**

Cervical cancer remains a major public health concern in Ethiopia because of limited screening and delayed diagnosis, resulting in high mortality. Most patients present with advanced-stage disease, predominantly squamous cell carcinoma. Despite the World Health Organization’s 90%–70%–90% strategy, screening coverage is low, and access to treatment remains inadequate. This study assessed the clinicopathologic characteristics and predictors of treatment uptake among cervical cancer patients managed at Jimma University Medical Center in southwest Ethiopia to inform evidence-based interventions.

**Methods:**

A cross-sectional study was conducted at Jimma University Medical Center, including all women with histologically confirmed cervical cancer between April 2019 and October 2024. Data on demographics, clinical presentation, pathology, and outcomes were collected using a pretested tool and entered into REDCap. Missing outcome data were supplemented through telephone interviews. Multiple imputation was used for missing explanatory variables (*n* = 20 datasets). Statistical analysis was performed using STATA version 17, univariable and multivariable logistic regression to identify predictors of treatment uptake.

**Result:**

Among the 719 women with cervical cancer, most were illiterate, grand multiparous, and from rural areas. Postmenopausal bleeding and pelvic pain were the most common presenting symptoms. Over two-thirds experienced delays in seeking care exceeding 90 days, and only 16% had a history of prior cervical cancer screening. Of the 709 patients with documented stage, 78.4% presented with advanced disease (stage IIB–IV)), while 21.6% were diagnosed at an early stage (stage I–IIA). Chemoradiotherapy was the main treatment modality, while surgery was rarely performed. Treatment uptake was higher among women younger than 55 years (OR = 2.27; 95% CI: 1.08–4.75) and those with formal education (OR = 3.31; 95% CI: 1.25–8.70), whereas women diagnosed at stage IV were significantly less likely to receive treatment (OR = 0.10; 95% CI: 0.03–0.43).

**Conclusion:**

These findings underscore the need to strengthen culturally appropriate community awareness and expand accessible screening programmes, particularly in rural areas, to improve timely treatment and outcomes for women in middle age. Vaccination and palliative care services should also be integrated into comprehensive cervical cancer care.

## Introduction

Cervical cancer (CC) is a major global health concern, ranking as the fourth most common cancer and the fourth leading cause of cancer-related deaths among women worldwide. In 2022, an estimated 604,127 new cases and 341,831 deaths were attributed to CC [1]. Notably, approximately 90% of cases occur in low- and middle-income countries (LMICs), where organised screening and human papillomavirus (HPV) vaccination programmes are often lacking [2]. In sub-Saharan Africa (SSA), the incidence of CC has been rising alarmingly [3], with Eastern and Southern Africa bearing the highest burden [4]. In Ethiopia, CC is the second most commonly diagnosed cancer among women, after breast cancer. It is also the second leading cause of cancer-related deaths in the country, with an estimated 7,445 new cases and 5,338 deaths annually [5].

The high mortality rate in Ethiopia is largely attributed to low screening coverage, stemming from a poorly coordinated CC screening programme [6, 7]. Late-stage diagnosis is common due to limited awareness, delays in diagnostic testing, and difficulties in accessing curative treatment [8–11]. However, HPV vaccination coverage in Ethiopia shows promise, with first- and second-dose coverage reported at 86% and 75%, respectively [5].

Understanding the histological types of CC is crucial for developing effective screening strategies. In regions where adenocarcinoma (AC) is more common, HPV DNA testing is important because routine cytology is less effective in detecting AC [12, 13]. Squamous cell carcinoma (SCC) is the predominant histological type in SSA and South-Central Asia, with a prevalence of 92.63% in SSA. By contrast, AC is relatively more common in North America and Europe, with a prevalence of 23.72% in Oceania [14]. A study in India found SCC to be the most prevalent histological type, accounting for 93.3% of cases, followed by AC at 5%, with other types making up 1.7% [15].

Similarly, SCC is the predominant histologic type in East African countries. In Uganda, SCC accounts for 86.3% of cases, with AC and other types comprising 13.7% [16]. In Malawi, SCC is reported in 85.7% of cases, followed by AC at 8.8% [17]. In Kenya, 91% of cases are SCC, 8% are AC, and 1% belong to other histologic types [18]. Studies in Ethiopia also indicate that SCC is the most common histologic type, with prevalence ranging from 83% to 96%, while AC accounts for 3.3% to 15% of cases [19–21].

Elimination of CC is possible, but many LMICs, including Ethiopia, lack effective preventive and therapeutic programmes. The World Health Organization urges global action to implement the 90%–70%–90% strategy by 2030: 90% of girls vaccinated by age 15, 70% of women screened at ages 35 and 45, and 90% of those diagnosed treated [22]. However, challenges in diagnosis and treatment in LMICs hinder this strategy because most patients in these regions present at a late stage due to inadequate early detection of precancerous lesions. In Ethiopia, 60.45% of patients are diagnosed at a late stage [23], largely because of limited awareness and knowledge about the disease, its risk factors, and screening methods, as well as restricted access to treatment [11].

Ethiopia’s CC prevention strategy includes tertiary care, but access to specialised treatments such as surgery, radiotherapy, and chemotherapy is limited to a few cities and remains costly. While early-stage cases are potentially curable, inadequate screening and limited access to treatment continue to hinder survival rates. The Ministry of Health aims to screen 80% of women aged 30–49 by 2025, yet national coverage was only 5% between 2015 and 2020 [24], highlighting significant challenges in achieving this goal.

Moreover, there is limited evidence on the clinicopathologic features and treatment uptake of patients with CC in Ethiopia, which is crucial for guiding policy decisions. This underscores the urgent need for updated data to support evidence-based interventions. The present study was performed to assess the clinicopathologic characteristics and predictors of treatment uptake among patients with CC managed at Jimma University Medical Center (JUMC), providing current evidence to inform policy and practice.

## Materials and methods

### Study design and setting

A hospital-based retrospective cross-sectional study was conducted among patients with CC who were registered at the Gynecology and Oncology Center of JUMC, Oromia, Ethiopia, between April 2019 and October 2024. Data were collected from June to November 2024. JUMC is located in Jimma Town, 350 km southwest of Addis Ababa, the capital of Ethiopia. It is the second tertiary teaching hospital in the country to provide comprehensive cancer treatment, including radiotherapy, since February 2022.

### Population, sample size, and sampling technique

The study included all patients with histologically diagnosed CC who were registered at JUMC during the study period.

### Data collection

The data extraction form was developed based on the study objectives and relevant literature, reviewed by senior clinicians and researchers, and pilot-tested on a subset of patient records. The finalized tool was implemented in REDCap with predefined variable names, value labels, validation rules, and branching logic to ensure data quality.

The dependent variable was treatment uptake, defined as initiation of any cervical cancer–directed treatment following histologic confirmation. Independent variables included sociodemographic, clinical, pathological, and health system–related factors. The instrument was used to collect sociodemographic information, clinical characteristics, pathological findings, and treatment initiation status. Trained female data collectors entered data into REDCap. Cancer registries from the Oncology Centre and Gynaecology Ward were used to obtain patients’ medical registration numbers, after which medical records were retrieved from the hospital archive.

For patients with undocumented outcomes, phone interviews were conducted with patients or caregivers. Overall, 200 records had incomplete outcome data; 158 patients were successfully contacted. During the phone interview, 69 patients were identified as deceased and were recorded accordingly, with no further interviews conducted. Outcome status was determined using medical records and caregiver reports where available.

### Statistical analysis

In this study, the health-seeking interval was defined as the period between the patient’s first recognition of symptoms and the first consultation with a healthcare provider. This interval was considered delayed if it exceeded 90 days. The diagnostic interval (DI) was defined as the time between the first consultation with a healthcare provider and the date of histologically confirmed diagnosis, and it was considered delayed if it exceeded 30 days. The pretreatment interval (PTI) was defined as the period between histopathologic diagnosis and commencement of cancer treatment, and it was considered delayed if it exceeded 30 days [16, 25–27].

Data were cleaned and analysed using STATA version 17 software. Descriptive statistics were used to summarize sociodemographic, clinical, pathological, and treatment characteristics. We examined sociodemographic, clinical, and health service–related characteristics as potential predictors of treatment initiation among women diagnosed with cervical cancer. These included age, place of residence, educational status of the patient and her husband, stage of cervical cancer, parity, and diagnostic interval. Treatment initiation was treated as a binary outcome (yes/no).

Because some predictor variables had missing values, multiple imputation with chained equations was carried out before conducting regression analyses to make the best use of the available data and reduce potential bias. Missing information was limited to the stage of disease and educational variables. These variables were imputed, while variables with complete data, including the outcome, were kept as observed. Categorical variables with more than two categories were imputed using multinomial logistic regression models, and all relevant predictors were included in the imputation process. Only twenty imputed datasets were generated, and a fixed random seed (rseed = 12345) was used to ensure reproducibility.

After imputation, we first performed univariable logistic regression analyses to explore crude associations between each predictor and treatment initiation. We then fitted a multivariable logistic regression model across the imputed datasets to identify independent predictors. Results were pooled using Stata’s mi estimate command and are presented as crude and adjusted odds ratios with 95% confidence intervals.

## Results

In total, 719 patients with CC were identified, and their data were accessed. The patients’ mean age was 52.6 ± 10.5 years (range, 28–96 years). Patients seeking care came from nearly all regions of the country, with the majority (69.9%) from the Oromia region. Most patients resided in rural areas (59.2%), were unable to read and write (43.5%), and were grand multiparous (71.1%). The most common clinical symptoms were postmenopausal bleeding (34.5%), persistent pelvic and/or back pain (26.1%), and abnormal vaginal discharge (16.5%). Approximately 66.5% of patients had a health-seeking interval longer than 90 days. For (6%) of the patients, the HIV status was positive, and only (16.0%) had a history of CC screening (Table 1). The median diagnostic interval was 20 days, and the median pre-treatment interval was 62 days (Table 2).

**Table 1:** Sociodemographic and clinical characteristics of patients with CC managed at JUMC, Ethiopia

Variable		Frequency	Percentage
Age, years (*n* = 719) Mean ± SD 52.6 ± 10.5	≤ 44	118	16.4
	45–54	303	42.1
	55–64	226	31.4
	≥ 65	72	10.0
Regions (*n* = 718)	Oromia	502	69.9
	Amhara	35	4.9
	Addis Ababa	37	5.1
	Gambela	13	1.8
	SNNP	126	17.6
	Others	5	0.7
Residence (*n* = 714)	Urban	291	40.8
	Rural	423	59.2
Educational status (*n* = 588)	Unable to read and write	256	43.5
	Primary	189	32.2
	Secondary and above	143	24.3
Education level of husband (585)	Unable to read and write	166	28.4
	Primary	182	31.1
	Secondary and above	237	40.5
Parity (*n* = 622)	≤ 1	17	2.7
	2–4	163	26.2
	≥ 5	442	71.1
Symptoms (*n* = 718)	Heavy and prolonged menstrual bleeding	181	13.8
	Contact bleeding	41	3.1
	Abnormal vaginal discharge	216	16.5
	Pain during intercourse	79	6.0
	Postmenopausal bleeding	453	34.5
	Persistent pelvic and or back pain	342	26.1
Health-seeking interval (*n* = 717) Median = 180 days	≤ 3 months	241	33.6
	> 3–12 months	386	53.8
	> 12 months	90	12.6
HIV status (*n* = 215)	Positive	13	6.0
	Negative	202	94.0
History of CC screening (*n* = 619)	No	520	84.0
	Yes	99	16.0

*SD* standard deviation, *SNNP* Southern Nations, Nationalities, and Peoples’ Region

**Table 2:** Time intervals for receiving services among patients with CC managed at JUMC, Ethiopia

Variables	Observations (*n*)	Median (IQR)	Min	Max
DI, days	578	20 (17.0)	1	809
PTI, days	521	62 (83.0)	3	1404
Death interval (treated patients), months	107	10.1 (10.1)	0.3	33.6
Death interval (untreated patients), months	30	6.6 (6.3)	1.6	35.1

*IQR* interquartile range, *Min* minimum, *Max* maximum

Of the 709 patients with documented International Federation of Gynecology and Obstetrics (FIGO) stage, half (50%) presented with stage IIB disease, and 78.4% had advanced-stage disease (stage IIB or above). SCC was the predominant histologic type (97.0%), followed by AC (2.7%). Approximately 92% of patients (91.6%, 95% CI: 89.3%–93.4%) received at least one form of treatment. Among those treated, 59.1% accessed services through the private wing, which required additional payment but did not require prior appointments. The most common treatment regimen was Chemoradiotherapy (CRT) (78.2%), while surgery was the least common (3.9%). Regarding patient outcomes, 53.0% were alive and 29.2% had died at the time of follow-up. The median time from histopathological diagnosis to death (death interval) was 10.1 months for treated patients and 6.6 months for untreated patients. The outcome for 17.8% of patients remained unknown. Among treated patients, 25% were cured, 36%% showed improvement, 5.9% experienced disease progression or complications, and 33.1% had died. By contrast, among untreated patients, 63.8% had died, and 36.2% were alive at the time of the interview (Tables 2 and 3).

**Table 3:** Stages, histopathology, and outcomes of patients with CC managed at JUMC, Ethiopia

Variable		Frequency	Percentage
Early-stage disease (*n* = 153/709)	Stage I	6	0.9
	Stage IIA	147	20.7
Advanced-stage disease (*n* = 556/709)	Stage IIB	355	50.0
	Stage III	156	22.0
	Stage IV	45	6.4
Histologic type (*n* = 707)	Squamous cell carcinoma	686	97.0
	Adenocarcinoma	19	2.7
	Adenosquamous cell carcinoma	2	0.3
Initiated on treatment (*N* = 719)	Yes	659	91.7
	No	60	8.3
Treatment regimen (*n* = 647)	Surgery	25	3.9
	Radiotherapy	29	4.4
	Chemotherapy	55	8.5
	Chemoradiotherapy	506	78.2
	Surgery with Chemoradiotherapy	32	5.0
Outcomes (all patients) (*n* = 586)	Alive	378	64.5
	Dead	208	35.5
Outcomes (treated patients) (*n* = 539)	Cured	135	25.0
	Improving	194	36.0
	Disease progression/complications	32	5.9
	Dead	178	33.1
Outcomes (untreated patients) (*n* = 47)	Alive	17	36.2
	Dead	30	63.8
Type of treatment service followed (*n* = 553)	Private wing	388	70.2
	Routine or scheduled	165	29.8

After multiple imputation, univariable logistic regression analyses showed that younger age, urban residence, higher educational status of both the patient and her husband, advanced stage of disease, high parity, and a prolonged diagnostic interval were associated with treatment initiation.

In the multiple imputation multivariable logistic regression model, younger age and higher patient educational status remained independent positive predictors of treatment initiation, whereas advanced stage of disease remained a strong negative predictor. Women under the age of 55 years had significantly higher odds of receiving treatment than did those aged 55 years and above (AOR = 2.23; 95% CI: 1.06–4.69), and women with primary education or above (formal education) were more likely to receive treatment than those without formal education (AOR = 3.31; 95% CI: 1.26–8.71). In contrast, women diagnosed with Stage IV cervical cancer were substantially less likely to initiate treatment (AOR = 0.11; 95% CI: 0.03–0.44). Although urban residence, husband’s educational status, high parity, and a prolonged diagnostic interval showed associations with treatment initiation in the adjusted analysis, these did not reach statistical significance (Table 4).

**Table 4:** Predictors of treatment initiation among patients with CC managed at JUMC, Ethiopia

Variable		Treatment initiation *N* = 557 (%)		Univariable logistic regression		Multiple imputation multivariable logistic regression	
		No	Yes	COR	95% CI	AOR	95% CI
Age, years	< 55	16 (4.92)	309 (95.08)	2.87	1.52, 5.39	2.23	1.06, 4.69
	≥ 55	30 (12.93)	202 (87.07)	1			
Residence	Urban	8 (3.29)	235 (96.71)	4.12	1.88, 9.00	2.10	0.78, 5.61
	Rural	38 (12.30)	271 (87.70)	1			
Education level of patient	Unable to read and write	38 (15.97)	200 (84.03)				
	Primary and above	8 (2.52)	309 (97.48)	3.92	2.12, 7.27	3.31	1.26, 8.71
Education level of husband	Unable to read and write	26 (17.11)	126 (82.89)	1			
	Primary and above	20 (5.00)	380 (95.00)	7.33	3.35, 16.06	2.23	0.99, 5.01
Stage of CC	Stage I–IIA2	8 (6.96)	107 (93.04)	1			
	Stage IIB	20 (7.41)	250 (92.59)	0.94	0.399, 2.19	1.21	0.48, 3.09
	Stage III	11 (8.80)	114 (91.20)	0.78	0.3, 2.0	0.82	0.28, 2.35
	Stage IV	7 (18.92)	30 (81.08)	0.32	0.11, 0.96	0.11	0.03, 0.44
Parity	≤ 4	7 (4.90)	136 (95.10)	1			
	≥ 5	39 (12.26)	279 (87.74)	0.37	0.16, 0.85	0.87	0.34, 2.22
Diagnostic interval	≤ 30 days	27 (8.88)	277 (91.12)	1			
	> 30 days	19 (16.81)	94 (83.19)	0.48	0.26, 0.91	0.54	0.26, 1.11

*COR* crude odds ratio, *AOR* adjusted odds ratio, *CI* confidence interval

## Discussion

This study investigated the clinicopathologic features and the predictors of treatment uptake among patients with CC managed at JUMC, Ethiopia. Although previous studies in Ethiopia have examined certain aspects of CC, they have not provided a comprehensive analysis, and no prior study has been conducted at this centre.

In this study of 719 patients, the majority were from rural areas, were unable to read and write, and were grand multiparous. The most commonly reported symptoms were postmenopausal bleeding and pelvic pain. Approximately two-thirds of patients experienced a health-seeking delay of more than 3 months, and only 16% had a history of prior screening. Among the 709 patients with documented FIGO stage, 78.4% were diagnosed at an advanced stage, with SCC accounting for 97.0% of cases. Most patients received treatment, predominantly CRT, while surgical intervention was rare. Multivariable analysis identified age, education level, and disease stage as significant predictors of treatment uptake. Patients younger than 55 years and those with formal education were more likely to receive treatment, whereas patients diagnosed at stage IV were significantly less likely to receive treatment than were those diagnosed at earlier stages.

The mean age of the patients with CC in this study (52.7 ± 10.6 years) is comparable to findings from a global analysis, in which the average age at diagnosis was 53 years [4], as well as studies in Uganda (50.0 ± 11.7 years) [16], a cancer centre in northwestern Nigeria (49.9 ± 11.9 years) [28], and Ghana (56.9 years) [29]. It is also similar to previous studies in Ethiopia, where the mean ages were 53 ± 12 years [19] and 52.6 ± 13.17 years [8]. However, it is higher than that reported in Malawi (44.9 years) [30]. The underlying age structure of the population influences the average age of patients. Additionally, such variations may be attributed to differences in risk factors influencing the progression of high-risk HPV infection to invasive CC, including HIV infection and other comorbidities. Notably, 47% of participants in the Malawian study were HIV-positive, compared with only 6% in the current study, which may have contributed to the earlier onset of invasive CC. The younger age at diagnosis in Malawi could also be associated with the high prevalence of sexual violence among children and adolescents in the country [31].

The majority of patients in this study came from rural areas (59.2%), comparable to a previous study (57.4%) [19]. Additionally, 43.5% of patients were unable to read or write, similar to findings from another study (38.5%) [8]. The consistency in sociodemographic characteristics across studies may indicate persistent disparities in CC prevention and control programmes among rural and uneducated populations.

The most common symptoms among patients in this study were postmenopausal bleeding (34.5%), persistent pelvic and/or back pain (26.1%), and abnormal vaginal discharge (16.5%). These patterns align with findings from Ghana, where postmenopausal bleeding, vaginal discharge, and pelvic pain accounted for 43.0%, 32.8%, and 7.0%, respectively [32]. The presence of postmenopausal bleeding should prompt immediate medical consultation unless cancer has already been ruled out. However, nearly two-thirds (66.4%) of patients in this study had a delayed health-seeking interval exceeding 3 months, compared with 15.2% in the Ghanaian study [32], 55.4% in a Moroccan study [26], and 23.4% in an earlier Ethiopian study [8]. These differences likely reflect variations in socioeconomic status, place of residence, and awareness of CC.

The median DI of 20 days in this study is not considered delayed, whereas the median PTI of 62 days indicates a significant delay. A similar PTI delay (2.4 months) was reported in a study conducted in Uganda [16]. This delayed PTI highlights the shortage of comprehensive treatment centres, particularly radiotherapy facilities, which are crucial for managing the majority of patients who present with advanced-stage disease.

Only 16% of patients in this study reported a history of CC screening, which is higher than the rate reported in Ghana [32] but markedly lower than the 57.4% observed in Botswana [33]. The higher screening coverage in Botswana may be attributed to the country’s relatively better socioeconomic conditions and its high HIV prevalence (69.7%) because individuals living with HIV are at increased risk of developing CC and are prioritised in screening programmes. These findings underscore the alarmingly low uptake of CC screening and highlight the urgent need to strengthen screening initiatives. Expanding access to globally recommended HPV DNA testing in culturally appropriate and accessible ways is essential to improving early detection and prevention of CC.

Among the 709 patients with documented FIGO staging in this study, 78.0% presented with advanced-stage disease (FIGO stages IIB–IV). This finding is consistent with studies from other African countries, where the prevalence of advanced-stage disease ranges from 70.6% in Ibadan, Nigeria, to 86% in Uganda [18, 28, 34–36]. However, it is notably higher than the reported prevalence in Southern Malawi (38.7%) [30] and in Ghana (66.0%) [37], yet slightly lower than that reported in an Ethiopian study, where the prevalence of late-stage or advanced-stage disease was 87.9% [38]. Variations in the prevalence of advanced-stage disease at diagnosis may be influenced by sociodemographic factors, awareness levels, and other contributing factors such as HIV prevalence. For instance, in Malawi, where 47% of patients with CC were HIV-positive, higher CC screening rates may have contributed to increased early-stage detection. The high proportion of advanced-stage cases in this study underscores the extremely low coverage of CC screening, limited knowledge and awareness about the disease, and inadequate access to early detection services.

SCC was the dominant histologic type (97.0%), exceeding the global average of 82.7%, while AC accounted for 2.7%, lower than the global estimate of 12.18% [14]. These findings are consistent with studies from India, where SCC comprised 93.3% and AC 5% [15], and from Ethiopia, where SCC accounted for 96% and AC 3% [9]. However, the prevalence of SCC was higher than that reported in Ghana (64.9% SCC, 8.4% AC) and Kenya (91% SCC, 8% AC) [16, 18, 29, 36, 37, 39], reflecting regional variations in histologic types, with SCC being more prevalent in East Africa. The higher prevalence of SCC is advantageous because it is more readily detected through screening and generally has better treatment outcomes than AC.

Approximately 92% of patients (91.6%, 95% CI: 89.3%–93.4%) initiated at least one form of cervical cancer–directed treatment. The majority (78.2%) received chemoradiotherapy, while only a small proportion (3.9%) underwent surgery. This treatment pattern is consistent with guideline-based management for advanced-stage disease and reflects the stage distribution in our cohort, where nearly four in five patients (78%) presented with advanced disease requiring combined chemotherapy and radiotherapy [40]. Nevertheless, this apparently high treatment uptake should be interpreted with caution. A substantial proportion of patients received treatment with palliative rather than curative intent, underscoring the consequences of late presentation. Moreover, treatment initiation and continuity were frequently compromised by prolonged waiting times related to limited radiotherapy capacity, as well as by the financial burden of cancer care, both of which contributed to treatment delays and non-compliance. These findings underscore the persistent challenge of late diagnosis in Ethiopia and are closely linked to the low coverage of cervical cancer screening, which remains markedly limited, with reported uptake ranging from 5% [24] to 14.8% [6, 7].

Among the 586 treated patients with known outcomes, 64.5% were alive, and 35.5% had died at the time of the interview. The median time from diagnosis to death was 10.1 months. The mortality rate in this study was higher than that reported in a previous Ethiopian study (23.5%) [41], likely due to differences in sociodemographic characteristics, including a higher proportion of rural patients and the predominance of advanced-stage disease in the present study.

In this study, multiple imputed multivariable analysis identified patient age, education level, and disease stage as significant predictors of CC treatment. Patients younger than 55 years and those with formal education were more likely to receive treatment, whereas those diagnosed at stage IV were significantly less likely to receive treatment than those diagnosed at earlier stages. This likely reflects the prioritisation of limited radiotherapy resources for women with earlier-stage disease.

Accordingly, women under the age of 55 years had significantly higher odds of receiving CC treatment than those aged 55 years and above (AOR = 2.23; 95% CI: 1.06–4.69). This finding is consistent with previous studies, which have also reported that younger patients are more likely to undergo treatment, although the specific age cut-offs vary across studies [42–48]. Several factors may explain this association. Younger women often exhibit greater health-seeking behaviour, leading to earlier diagnosis and better engagement with treatment services. In addition, they typically have fewer comorbidities, higher Eastern Cooperative Oncology Group performance status scores, and more robust immune function, which may increase their likelihood of being eligible for, and successfully completing cancer treatment.

Similarly, women with formal education were significantly more likely to receive CC treatment than were those without formal education (OR = 3.31; 95% CI: 1.26–8.71). This association may be attributed to the greater health literacy and awareness commonly observed among educated women, which can enhance their understanding of the disease, available treatment options, and the importance of timely care. Previous studies have likewise shown that women with formal education are more likely to engage with health services, adhere to medical advice, and navigate the healthcare system effectively, all of which contribute to increased treatment uptake [49–52].

Furthermore, in this study, women diagnosed with stage IV CC were significantly less likely to receive treatment than were those diagnosed at earlier stages (I–IIA2) (AOR = 0.11; 95% CI: 0.03–0.44). This finding is consistent with previous studies demonstrating that women with advanced-stage disease are more likely to present late and less likely to initiate treatment [53–55]. This may be because stage IV CC often involves metastasis to distant organs, limiting the effectiveness of curative treatment options. Consequently, healthcare providers may prioritise palliative care over definitive treatment. In addition, some patients may choose not to initiate treatment because of the perceived limited benefits and potential adverse effects associated with advanced therapies.

### Strengths and limitations

This study provides a comprehensive evaluation of patients with CC by examining sociodemographic characteristics, delays in health-seeking and diagnostic intervals, clinicopathologic features, and predictors of treatment initiation among those diagnosed during the study period. However, a key limitation lies in the retrospective nature of treatment outcome assessment. Because of connectivity challenges and the unavailability of accurate contact information, some patients could not be reached by telephone, resulting in an undetermined treatment status for a subset of the study population.

## Conclusion

This study highlights substantial delays in the diagnosis and treatment of CC in Ethiopia, with most patients presenting at advanced stages, largely because of low screening uptake and prolonged health-seeking intervals. Treatment initiation was significantly associated with age, education status, and stage at diagnosis. These findings underscore the urgent need to strengthen community awareness, expand access to CC screening services, and implement effective early detection strategies to enable timely treatment and improve patient outcomes.

## Data Availability

The datasets used and/or analyzed during the current study are available from the corresponding author on reasonable request.

## Abbreviations

CC: Cervical cancer
LMICs: Low- and middle-income countries
SSA: Sub-Saharan Africa
AC: Adenocarcinoma
SCC: Squamous cell carcinoma
FIGO: International Federation of Gynecology and Obstetrics
DI: Diagnostic interval
PTI: Pre-treatment interval
HPV: Human papillomavirus
JUMC: Jimma University Medical Center
